# Judgments of Learning for Words in Vertical Space

**DOI:** 10.3389/fpsyg.2016.01894

**Published:** 2016-12-01

**Authors:** Karlos Luna, Beatriz Martín-Luengo, Yury Shtyrov, Andriy Myachykov

**Affiliations:** ^1^Psychology Research Centre, University of MinhoBraga, Portugal; ^2^Centre for Cognition and Decision Making, National Research University Higher School of EconomicsMoscow, Russia; ^3^Department of Clinical Medicine, Center of Functionally Integrative Neuroscience, Aarhus UniversityAarhus, Denmark; ^4^Department of Psychology, Northumbria UniversityNewcastle upon Tyne, UK

**Keywords:** embodied cognition, metamemory, judgments of learning, vertical location, Bayesian analyses

## Abstract

Close relationship between physical space and internal knowledge representations has received ample support in the literature. For example, location of visually perceived information in vertical space has been shown to affect different numerical judgments. In addition, physical dimensions, such as weight or font size, were shown to affect judgments of learning (JOLs, an estimation of the likelihood that an item will be remembered later, or its perceived memorability). In two experiments we tested the hypothesis that differences in positioning words in vertical space may affect their perceived memorability, i.e., JOLs. In both Experiments, the words were presented in lower or in upper screen locations. In Experiment 1, JOLs were collected in the centre of the screen following word presentation. In Experiment 2, JOLs were collected at the point of word presentation and in the same location. In both experiments participants completed a free recall test. JOLs were compared between different vertically displaced presentation locations. In general, Bayesian analyses showed evidence in support for the null effect of vertical location on JOLs. We interpret our results as indicating that the effects of physical dimensions on JOLs are mediated by subjective importance, information that vertical location alone fails to convey.

## Introduction

How are objects and concepts processed and represented? Recent literature highlights the connection between real-world physical properties, dimensions, and references and the internal representations, most evident in the cognitive concepts of “grounding” or “embodiment” (Wilson and Golonka, 2013; Krishna and Schwarz, 2014). For example, various aspects of number processing and mental arithmetic, such as detection, random number generation, counting, or pointing were all shown to depend on spatial-numerical mapping (for recent reviews, see Fischer and Shaki, 2014 and Myachykov et al., 2014). One of such mappings is reflected in various effects of displacement along the vertical axis on behavioral responses. For example, positive words are identified faster when presented above fixation, while negative words are identified faster when presented below fixation (Meier and Robinson, 2004). This result is explained as reflecting associations of upper space with positive, and lower space with negative valence (Meier and Robinson, 2004, 2006). An extreme case of this is the vertical mapping of the divine and infernal: God is up and the devil is down (Meier et al., 2007). Another example of the effect of vertical space on conceptual processing is that more powerful agents are identified faster when presented in upper space, suggesting that power is also associated with vertical space (Schubert, 2005). Finally, vertical location of a stimulus was also linked to physical features of a sound: louder tones are associated with the upper location (Puigcerver et al., 2016). These and similar studies consistently point to the existence of a regular spatial component in abstract concept representations, and this component seems to have scalar properties (e.g., more-less, better-worse, heavier-lighter).

While the studies reviewed above investigated the emergence of spatial-conceptual mappings from online perceptual input, other studies suggest that spatial-numerical coding continues to be an intrinsic property of representations stored oﬄine (Abrahamse et al., 2016). If so, spatial-conceptual associations may be assumed to be relevant for perception and for memory encoding, retrieval, and maintenance. Supporting this claim, some studies showed that physical dimensions affect the estimations of the likelihood that a studied word will be successfully recalled later (judgments of learning, or JOLs). JOLs are subjective estimations made by the agent, usually immediately after encoding an item or soon after. They are one of the most popular measures of metamemory (for a review, see Metcalfe and Dunlosky, 2008). Besides the theoretical interest of JOLs for a study of the factors affecting memorability, they also have an applied value, particularly in educational settings. For example, JOLs are used to determine which item will be studied next (i.e., restudy decisions, Nelson et al., 1994; Kimball et al., 2012), or when to stop studying (Metcalfe and Kornell, 2005).

Importantly, JOLs were also shown to be affected by the stimulus’ physical dimensions, supposedly unrelated to efficient memory consolidation. For example, words studied when carrying a heavier object are rated as more memorable (i.e., higher JOLs) than when carrying a lighter object (Alban and Kelley, 2013). The authors explained this result because weight embodies the concept of importance. Heavier objects are rated as more important (Jostmann et al., 2009; Ackerman et al., 2010), and participants believe that more important items will be better remembered. Other physical dimensions, such as font size and loudness, were also shown to affect JOLs. For example, words presented in a larger font are considered as more memorable (i.e., rated with higher JOLs) than words presented in a smaller font size (Rhodes and Castel, 2008; Mueller et al., 2014). Similarly, auditory words presented in louder volume are rated with higher JOLs, and, because of the relationship between JOLs and restudy decisions (the decision about whether to restudy an item again or not), participants also rated words presented louder with lower restudy decisions (Rhodes and Castel, 2009).

In sum, physical dimensions seem to affect JOLs, but, critically, they do not affect actual memory performance (Rhodes and Castel, 2008; Alban and Kelley, 2013). This is important because, for example, a student reading from a heavy textbook seated in her lap may think that she has learnt the content better than she actually has and move to another unit. Therefore, the identification of factors that may affect memorability but not memory performance is crucial in educational settings.

The main objective of this research was to explore one physical dimension that may affect JOLs but not memory performance. In particular, we studied the relationship between the spatial location (in particular, vertical location) of a to-be-remembered word and the subjective ratings of how memorable that word is (i.e., JOLs). We expected that the words presented above central fixation would be rated with higher JOLs than the words presented below. As the research reviewed above shows, different research lines converge to support our hypothesis. First, there is a relationship between vertical space and word valence, with words presented up in the screen perceived as generally more positive, good, and powerful (Meier and Robinson, 2004, 2006). This, in turn, may increase their *perceived* memorability and lead to higher JOLs. Similarly, information presented higher in a text or on a screen is typically more important (e.g., the title in an article, news headlines, or the president or CEO in a company’s organizational chart). The upper locations, then, may be considered as more important and relevant and, therefore, information presented there may be rated as more memorable. Finally, our hypothesis is motivated by the research on the mental number space, a popular explanation for the spatial-numerical mapping (Fischer and Shaki, 2014). For vertical location, the mental number space proposes that we have a linear mental representation, with larger magnitudes located on top of smaller magnitudes (Ito and Hatta, 2004; Shaki and Fisher, 2012). This vertical mental line may increase any numerical judgment made on items presented higher up over those presented lower down.

We also expected that the vertical positioning will affect restudy decisions. There is a strong inverse relationship between JOLs and restudy decisions (Nelson et al., 1994; Luna et al., unpublished): the higher the JOL, the lower the willingness to restudy a given item later. If words presented up in space are rated with higher JOLs, then we predicted that they will be rated with lower restudy decisions than words presented down. Finally, we did not expect word location to affect memory performance *per se*. To test these hypotheses, we conducted two experiments in which words were presented either in the upper or the lower parts of the screen and JOLs, restudy decisions (Experiment 1), and memory performance measures were collected. To analyze the data, we applied Bayesian analysis that has the major advantage over classic null hypothesis significance testing (NHST) in that it can provide evidence in support of the null hypothesis itself as well. We will briefly introduce this technique below.

### Statistical Analyses

To test our hypotheses we ran Bayes-factor analyses using JASP (JASP Team, 2016) and the package BayesFactor (Morey and Rouder, 2015) in R (R Core Team, 2016). Bayesian analyses can provide evidence in support of either the null or the alternative hypothesis, and has been proposed as an alternative to standard NHST (Wagenmakers, 2007; Feinberg and Gonzalez, 2012; Kruschke, 2013). For a basic understanding of Bayesian analysis and the associated computations, see Wagenmakers et al. (2016). For a more in-depth explanation for social scientists, see Kruschke (2015).

In a nutshell, the Bayes factor (henceforth *BF*) allows updating the beliefs about the data with evidence collected after the analysis. For example, if the null hypothesis (*H*_0_) is that *M*_1_ = *M*_2_, and the alternative hypothesis (*H*_1_) is that *M*_1_ ≠ *M*_2_, a *BF* = 3 shows moderate evidence in favor of *H*_1_. In other words, we had a prior belief that *M*_1_ = *M*_2_ (*H*_0_). However, after the observation of the data we have to update that belief because it is three times more likely that *M*_1_ ≠ *M*_2_ than *M*_1_ = *M*_2_. A usual concern comes with the cut-off points to decide what should be considered moderate or strong evidence. Here we will follow the recommendations of the JASP Team (2016): a *BF* of 1 shows no evidence in support of either hypothesis. Evidence accumulates in favor of *H*_1_ when *BF* increases and in favor of *H*_0_ when it decreases. A *BF* from 1 to 3 is considered anecdotal evidence in favor of *H*_1_, from 3 to 10 is moderate evidence, from 10 to 30 is strong, and more than 30 shows extreme evidence in support of *H*_1_. A *BF* from 0.33 to 1 shows anecdotal evidence in support of *H*_0_, from 0.10 to 0.33 is moderate evidence, from 0.03 to 0.10 is strong evidence, and lower than 0.03 is considered extreme evidence in support of *H*_0_. A *BF* of 0.20 can be interpreted as above by computing the inverse (1/0.20 = 5). Thus, a *BF* of 0.20 in favor of *H*_0_ can be interpreted as that the likelihood that *M*_1_ = *M*_2_ is true is five times higher than *M*_1_ ≠ *M*_2_. It is also important to note that the above limits are not exact cut-off thresholds, but rather interpretation guidelines. For example, *BF* = 2.9 should not be interpreted in a very different way than *BF* = 3.1, despite corresponding to a different label. In the analyses presented below, the null hypothesis (*H*_0_) was that *M*_Up_ = *M*_Down_, and the alternative hypothesis was that *M*_Up_ ≠ *M*_Down_.

In addition to Bayesian analyses, in the experiments below we also report Student’s *t-*tests and *Cohen’s d* (henceforth *d*) as an effect size measure for completeness. The comparisons were always up vs. down, and thus negative *d′*s indicate higher values for words presented down. Even though location was a within-subject manipulation, we computed *d′*s with the formula for between-subjects manipulations, as suggested by Cumming (2012, pp. 291–292).

## Experiment 1

We based this study on the typical experimental set up for a computerized study in which a list of words is presented and JOLs are collected (e.g., Rhodes and Castel, 2008).

### Method

#### Participants and Design

Twenty-four participants (two males, age *M* = 20.67 years old, *SD* = 3.94; all native Russian speakers) completed the experiment in exchange for a monetary compensation. Participants were randomly assigned to one of the two counterbalanced conditions. The only independent variable was the word location (upper or lower), manipulated within subjects.

Experimental protocols in both experiments adhered to the Helsinki Declaration and were approved by the Psychology Department Research Ethics Committee, Higher School of Economics. Informed written consent was obtained from all participants.

#### Materials

To create the materials for the experiment we selected 44 Russian nouns from an open corpus repository^1^, four to be used as primacy and recency buffers and 40 target words (see Supplementary Materials for the full list of items). To control for linguistic properties, we selected nouns with word frequency between 40 and 80 per million and between 5 and 8 letters. Any words with emotional connotation (e.g., happiness, murder) were excluded to avoid potential confounds related to differences between neutral and emotional words (Kensinger, 2009). Also, words with a clear spatial reference (e.g., sky, foot) were removed to avoid any interference of the internal representation of the referent of the word with the physical location in which the word was presented. For counterbalancing purposes, target words were divided into two subsets of 20 with matched frequency and number of letters.

#### Procedure

Participants completed the experiment individually on a computer with a 21.5″ monitor screen. The experiment was programmed in LiveCode (2015). After providing written consent and basic demographics, participants were shown a screen with the experimental instructions. They were instructed to maintain a central fixation until a word would be presented in different parts of the screen. They were instructed to read and remember the word and provide a JOL for each individual word as well as a restudy decision. The instructions also mentioned that, after the words, there would be a memory test. For JOLs, the instructions prompted participants to “indicate on a scale from 0 to 100% your confidence that you will be able to remember the word later. If you are completely certain that you will not remember the word later, then select 0. If you are completely sure that you are going to remember the word later, then select 100.” For restudy decisions, the instructions requested to “indicate if you would like to see the word at a later time to help you remember it” in a yes/no format. The words were never actually repeated. Participants read the instructions at their own pace.

The sequence for each of the 44 words was as follows. First, a fixation cross (+) was presented in the centre of the screen for one second. After it, the first word was presented for 3 s. Words were presented in the centre of the horizontal axis, half of them in the upper part of the screen and half in the lower part, counterbalanced. Participants sat at approximately 70 cm viewing distance from the monitor, and the visual angle between the upper and lower word location was approximately 20 visual degrees (11.5 cm of distance from the fixation point to the upper or lower locations). After each word and in a different screen, participants provided JOLs in a scale from 0 to 100 in deciles, and the restudy decisions in a yes/no format. Both JOLs and restudy decisions were displayed at the centre of the screen. There was no time limit to provide the responses.

Following the study phase, participants completed simple arithmetic operations for 4 min. The objective of this task was to introduce a delay and avoid rehearsal, so during the test we measured contents no longer in short-term memory. A criterion of accuracy higher than 75 percent in the arithmetic operations was set to guarantee that participants did not try to rehearse the words during the delay. All the participants met the criterion (accuracy range: 86.49–100 percent). Finally, in the memory test phase, participants were given 4 min to write down all the words that they remembered from the study phase. When the time was over, participants were debriefed and dismissed.

### Results

Main statistics of Experiment 1 are presented in **Table 1**.

**Table 1:** Judgments of learning (JOLs), restudy decisions, and free recall results for words presented up and down in Experiment 1: mean (standard deviation) [95 percent confidence interval] and the Bayes Factor (BF) of the comparison between both conditions.

	Words presented Up	Words presented Down	BF
JOLs	74.60 (21.37) [65.58, 83.63]	76.06 (20.98) [67.20, 84.92]	0.645
Restudy decisions	9.58 (19.83) [1.21, 17.96]	11.04 (21.26) [2.06, 20.02]	0.492
Memory performance	31.25 (20.23) [23.16, 39.34]	32.71 (23.17) [23.44, 41.98]	0.258

#### Judgments of Learning

A *BF* = 0.645 showed anecdotal evidence in favor of no differences between the means. In particular, the belief that JOL means are similar is 1.55 times higher after the observation of the data than before. This result should be interpreted as inconclusive. The Student’s *t-*test showed that there were indeed no statistically significant differences between JOLs for words presented in upper or lower locations, *t*(23) = –1.59, *p* = 0.125, *d* = –0.07.

#### Restudy Decisions

The results here mimicked those for the JOLs. A *BF* = 0.492 showed anecdotal evidence in support of no differences between conditions, and Student’s *t-*test again showed no statistically significant differences, *t*(23) = –1.37, *p* = 0.183, *d* = –0.07.

#### Memory Performance

A *BF* = 0.258 showed moderate evidence in favor of no differences. The belief that there were no differences between means was 3.87 times higher after the observation of the data, which allow us to conclude that we found evidence that the word location did not affect memory performance. The Student’s *t*-test also showed no differences, *t*(23) = –0.64, *p* = 0.529, *d* = –0.06, although it is important to remember that, unlike Bayesian analysis, a NHST test that fails to reject the alternative hypothesis cannot be interpreted as providing support for the null hypothesis.

### Discussion

The results of Experiment 1 showed that our main manipulation did not affect any of the dependent measures, which was confirmed by both Bayesian statistics and more conventional tests. Although not completely conclusive, these findings suggest that vertical location may not affect memorability, or the restudy decisions. Our results also suggest that vertical location did not affect memory performance.

One reason why the experiment failed to find an effect of vertical location on JOLs could be that we collected JOLs *after* the presentation of the words (i.e., oﬄine), as done usually in studies that collect that measure. If, however, the effect of word location on JOLs is short lived, it may have already faded by the time the JOL was collected. In addition, for a more controlled experimental set up in Experiment 1 JOLs were collected in the centre of the screen. If, as hypothesized, presenting words in the upper part of the screen activates the mental space embodied in our cognitive system, then collecting JOLs in the centre of the screen may have reduced their activation effectively working against the predicted pattern. Therefore, to overcome these confounds and further test these ideas, we conducted Experiment 2, in which we collected JOLs while the word was on the screen (i.e., online) and in the part of space congruent with the presentation of the word, e.g., up or down.

## Experiment 2

### Method

#### Participants and Design

Twenty-four new participants (six male, age *M* = 21.04 years old, *SD* = 3.90, native Russian speakers) took part in the experiment in exchange for a small remuneration. The design was the same as in Experiment 1.

#### Materials and Procedure

The same materials as in Experiment 1 were used (see Supplementary Materials), but several changes were introduced in the procedure. After the central fixation point, the word was presented in either the upper or the lower part of the screen. Next to the word, there was an empty text field so that participants could enter the JOL in a numeric format from 0 to 100. The text box appeared either immediately to the right or to the left of the word, counterbalanced across conditions. The word was displayed on the screen until participants rated the JOL and pressed the “enter” key. Therefore, another major difference with Experiment 1 was that words were displayed for a variable time decided by the participant. This was done to prevent participants from not providing ratings for all the words. To keep the screen as clean as possible in this modified presentation mode, restudy decisions were not collected, but the actual study times for each word were recorded instead. After participants pressed the “enter” key, the fixation point-word cycle started again. The filler task and the memory test followed identical to Experiment 1.

### Results

Preliminary analyses showed that the location of the JOL, to the right or to the left of the word, did not affect any of the measures nor interacted with other variables. Main statistics of Experiment 2 are presented in **Table 2**.

**Table 2:** Judgments of learning, study time, and free recall results for words presented up and down in Experiment 2: mean (standard deviation) [95 percent confidence interval] and the Bayes Factor (BF) of the comparison between both conditions.

	Words presented Up	Words presented Down	BF
JOLs	56.48 (24.64) [46.62, 66.34]	57.06 (24.46) [47.28, 66.85]	0.230
Study time	4500 (3150) [3239, 5760]	4409 (3302) [3088, 5730]	0.253
Memory performance	17.29 (17.94) [10.12, 24.47]	21.25 (16.89) [14.49, 28.01]	0.719

#### Judgments of Learning

A *BF* = 0.230 showed moderate evidence in favor of no differences between means. The belief that JOL means are similar is 4.34 times higher after the observation of the data than before. The Student’s *t-*test showed that the difference between JOLs for words presented in upper or lower location was not statistically significant, *t*(23) = –0.39, *p* = 0.702, *d* = –0.02.

#### Study Time

For each participant, study times above 3 and below -3 z-scores were considered outliers and removed. Only 18 outliers were identified (2 percent of the responses). Analyses were conducted as usual with the remaining responses. A *BF* = 0.253 showed moderate evidence in support of no differences between conditions (3.95 times higher after than before data). The Student’s *t-*test was not significant, *t*(23) = 0.60, *p* = 0.553, *d* = 0.03.

#### Memory Performance

A *BF* = 0.719 showed anecdotal evidence in support of no differences between the means (1.39 times higher after than before the data). The Student’s *t-*test was also not significant, *t*(23) = –1.67, *p* = 0.108, *d* = –0.22.

### Discussion

In Experiment 2 we collected JOLs online, i.e., during study, and in the same vertical location as the target word. The results showed evidence against the hypothesis that vertical location may have an effect on memorability, i.e., our results suggest that there is no effect of vertical space on memorability. In addition, our results also suggest that vertical location has no effect on study time or on memory performance.

## General Discussion

In two experiments we tested the hypothesis that vertical location may affect perceived memorability of studied words. Despite the extensively reported effects of the vertical displacement and spatial properties of a stimulus on aspects of semantic processing and different stimulus-associated judgments, including effects of physical dimensions on JOLs, we failed to confirm this hypothesis. Importantly, unlike most experimental psychology studies that use conventional null hypothesis statistical testing, we obtained statistical evidence in support of the null hypothesis by the use of Bayesian analyses, which have a marked advantage of providing evidence for and against both alternative and null hypotheses.

One explanation for our results is that the effect of spatial location may be limited to automatic processes. For example, studies on the spatial-numerical association of response codes (SNARC; Dehaene et al., 1993) show that small numbers are processed faster and with higher accuracy when the response is produced by pressing a button located to the left or down, and larger numbers are processed faster and more accurately when the button is to the right or up. To successfully perform on this simple task, participants need to detect and apprehend one of the properties of the number (e.g., parity) and press a key with the left or right hand according to a given rule. This does not require extensive processing of the stimulus, while making a JOL is a deliberative process based on the monitoring of memories, and on the projected performance. As such, a JOL is a construct that involves many complex factors including the knowledge about how our personal memory work (e.g., Do I usually remember this type of material?), and the beliefs about how memory works in general (e.g., I know that after some time I will forget part of the information). Even some physical dimensions unrelated with actual memory, such as weight and font size, are taken into account to make a JOL. Hence, one possibility is that spatial location only affects fast and automatic processes where there is no extensive processing of the stimuli or of the nature of the associated response, while heavy involvement of controlled processing stages cancels or masks any putative effects.

Despite this, there are well documented effects of other physical dimensions on a deliberative response such as JOLs. Their effect on JOLs seems to be mediated by the perception of how important the item is. For example, weight was shown to affect JOLs because heavy objects are perceived as more important (Alban and Kelley, 2013). Similarly, font size may also embody the idea of importance, which may explain why people simply believe that words in larger font will be better remembered (Mueller et al., 2014). We also hypothesized that vertical location may convey information about importance, but our results did not support that idea. As such, our findings suggest that there is little association between vertical space and perceived importance, at least for the subjective measurements such as JOLs. Future research is necessary to further explore these relationships at different processing levels. Last but not least, such future research will benefit from adding Bayesian statistics to their methodological inventory, as it appears a powerful tool that can add valuable information to the more conventional statistical tests.

## Author Contributions

All authors contributed to the idea and design of the experiments. KL programmed the experiments and BM-L collected data. KL and BM-L analyzed the results. KL wrote a first draft and all the authors revised and contributed to the final version.

## Conflict of Interest Statement

The authors declare that the research was conducted in the absence of any commercial or financial relationships that could be construed as a potential conflict of interest.

## Footnotes

1 http://corpus.leeds.ac.uk/serge/frqlist/rnc-modern.num.html
