# Supplementary material for: Judgments of Learning for Words in Vertical Space
Source: Front Psychol. 2016 Dec 1;7:1894. doi: 10.3389/fpsyg.2016.01894 (PMC5131559; doi:10.3389/fpsyg.2016.01894)
Supplement: Supplementary file 1 [file Data_Sheet_1.docx]

**Supplementary Materials**

List of critical items used in Experiments 1 and 2. The experiments were conducted in Russian language. Original Russian words and their English translation are provided. Different columns show items in different counterbalance conditions.

| **Russian (original)** | **English translation** | **Russian (original)** | **English translation** |
| --- | --- | --- | --- |
| адрес | address | автобус | bus |
| выпуск | issue | аспект | aspect |
| гарантия | guaranty | беседа | conversation |
| дворец | castle | ведение | preface |
| деревня | village | ветер | wind |
| животное | animal | вещество | matter |
| контакт | contact | карман | pocket |
| коридор | hallway | кровать | bed |
| костюм | suit | памятник | monument |
| одежда | clothes | питание | nutrition |
| пакет | packet | поставка | delivery |
| поворот | turn | прибор | device |
| покупка | purchase | ремонт | repair |
| портрет | portait | рождение | birth |
| препарат | medical drug | секрет | secret |
| просьба | request | сектор | sector |
| рисунок | drawing | сосед | neighbour |
| стадия | stage | стандарт | standard |
| сущность | essence | существо | creature |
| фигура | figure | эпоха | era |

Buffer items:

| **Russian (original)** | **English translation** |
| --- | --- |
| версия | version |
| инфекция | infection |
| корпус | building |
| ожидание | expectation |
